# Božo Kralj (1932–2020): in memoriam

**DOI:** 10.1007/s00192-020-04656-z

**Published:** 2021-01-13

**Authors:** Adolf Lukanović, David Lukanović

**Affiliations:** 1 Department of Gynecology, Division of Gynecology and Obstetrics, Ljubljana University Medical Center, Zaloška 2, 1000 Ljubljana, Slovenia; 2 Department of Gynecology and Obstetrics, Faculty of Medicine, University of Ljubljana, Šlajmerjeva 3, 1000 Ljubljana, Slovenia

Horace once said *Exegi monumentum aere perennius*: “I have created a monument more lasting than bronze.” Božo Kralj’s monument is made of another, much nobler, substance: a noble memory, a memory that remains. The greatness of a man is measured by his legacy. Kralj’s legacy is his invaluable work in urogynecology. And it remains a model that invites imitation.

**Fig. 1:**
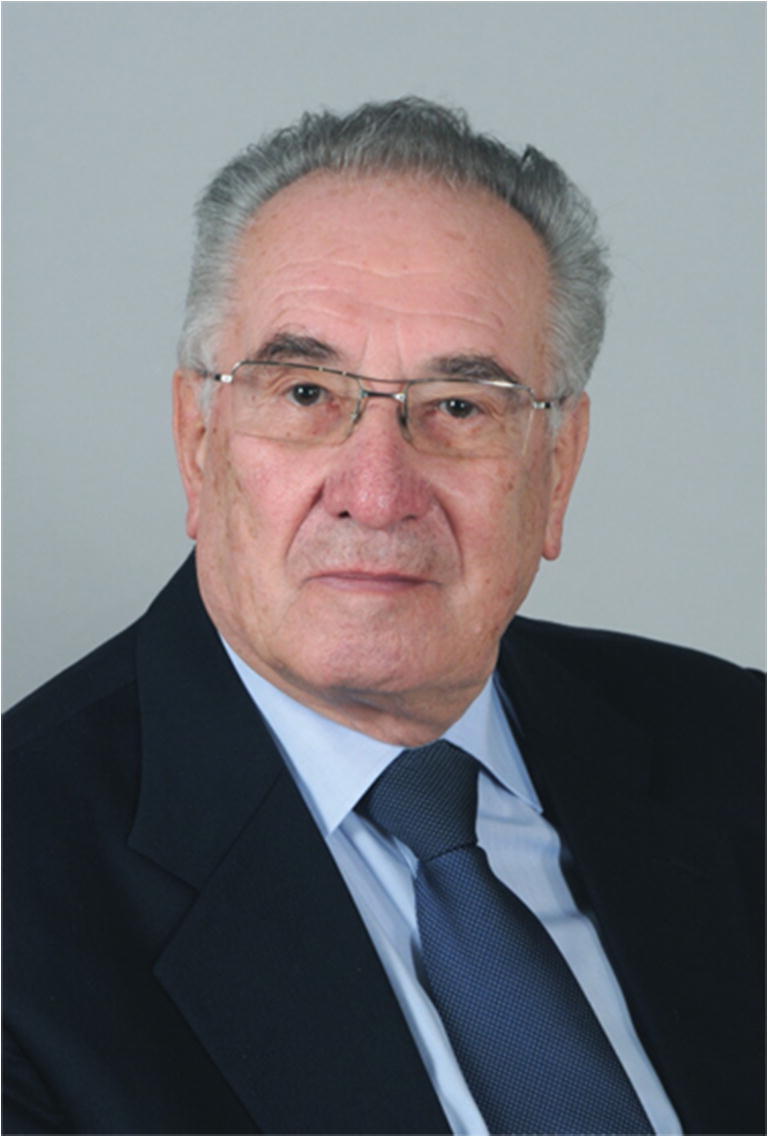
Božo Kralj, 80th birthday

We only become fully aware of the importance and nobility of our human relationships and friendships when we are affected by the cruel reality of losing someone close to us, a person to whom we are bound by memories and emotions. On 22 October 2020, after an insidious illness, Božo Kralj, MD, professor, and gynecology and obstetrics specialist, passed away (Fig. 1).

A recognition of the truth that death is the ultimate limitation among all those placed upon humankind, both in youth and in old age, causes us to reflect on our respect for the life and memory of the deceased. Saying farewell is always painful, especially if it is final. When we have to bid farewell forever to a man who has stood by our sides for many years, even decades, as an indispensable colleague, a respected teacher, and a loyal friend, such a farewell is especially bitter. The personal virtues that distinguished Božo Kralj, such as his versatile professional striving and perseverance at work, accompanied by his eagerness to keep learning new things, soon led him to professional recognition abroad. He contributed to the exceptional development of Slovenian gynecology in recent decades, left an immeasurable mark on urogynecology worldwide, and contributed to the international acknowledgement and stature of the Slovenian profession abroad.

Božo was born on 7 May 1932, in Maribor. He graduated from the Faculty of Medicine in Ljubljana in 1956 and completed his specialization in gynecology and obstetrics in 1965, when he began working at the Ljubljana University Medical Center (UMC) Gynecology and Obstetrics Division, where he remained until his retirement in 1998. From 1992 to 1998 he served as the medical director of the UMC Gynecology and Obstetrics Division. Early in his work he had already become aware of the importance of lifelong learning and sharing professional knowledge with his younger colleagues. He rounded out his teaching career in 1990, when he was named a full professor of gynecology and obstetrics at the University of Ljubljana, Faculty of Medicine.

Božo began his career studying cervical insufficiency, then dealt in-depth with urological problems after the treatment of cervical cancer and developed guidelines for the treatment of pelvic inflammatory disease and septic abortion. From 1969 onward, he developed the new interdisciplinary field of urogynecology at the Ljubljana University Medical Center Gynecology and Obstetrics Division, implemented functional electrical stimulation for the conservative treatment of urinary incontinence, and introduced urodynamic testing in clinical practice into many hospitals throughout Slovenia.

**Fig. 2:**
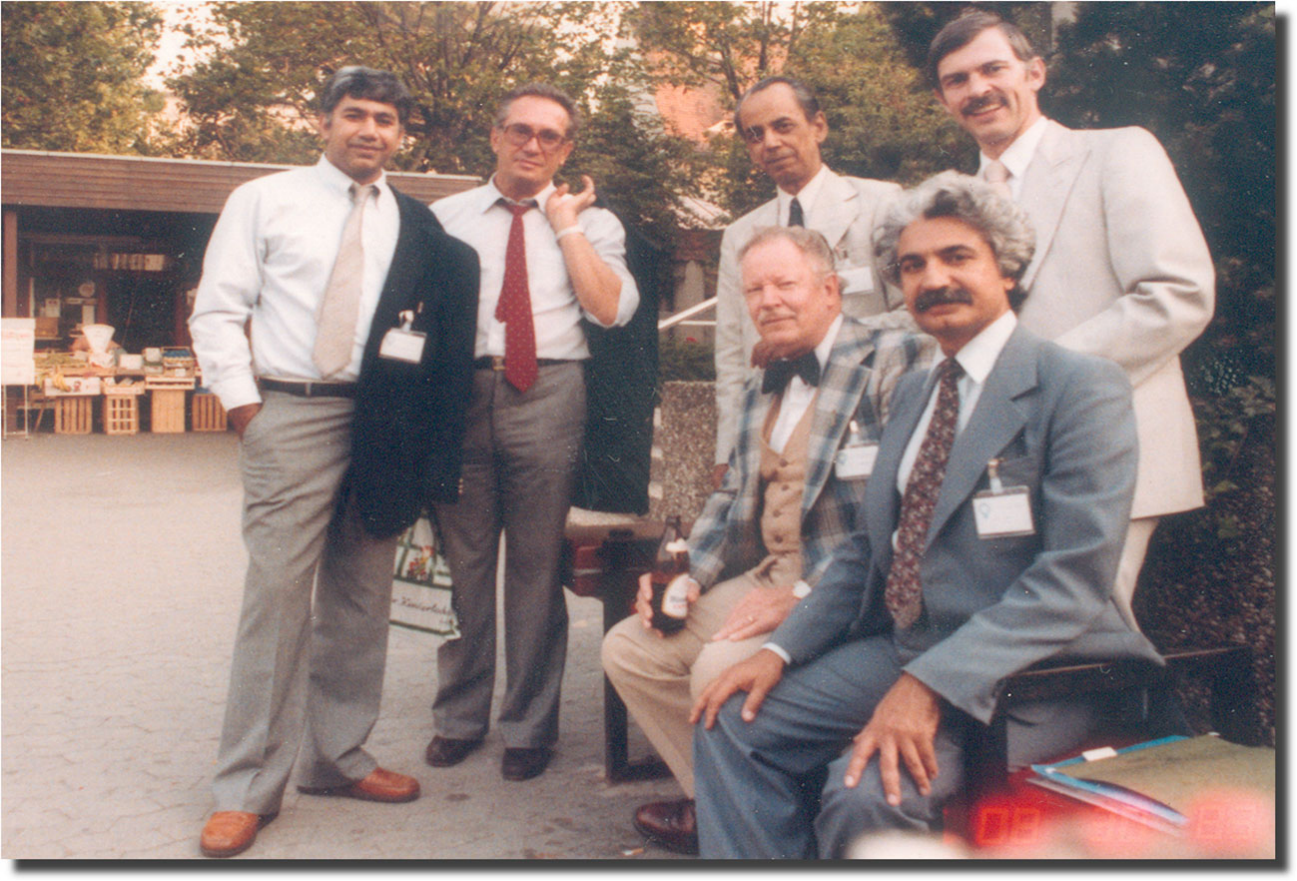
ICS 1983, Aachen, Germany: Božo Kralj is second from left among the urogynecology pioneers

His awareness of the extent of urinary incontinence problems among women led him to become one of 11 founding members of the International Urogynecology Association, established in 1976 at the Federation of Gynecology and Obstetrics (FIGO) conference in Mexico City. He was the International Urogynecological Association (IUGA) vice president from 1985 to 1988 and president from 1988 to 1990. In 1987, he chaired the IUGA’s 12th annual meeting, held in Ljubljana, Slovenia (Fig. 2).

**Fig. 3:**
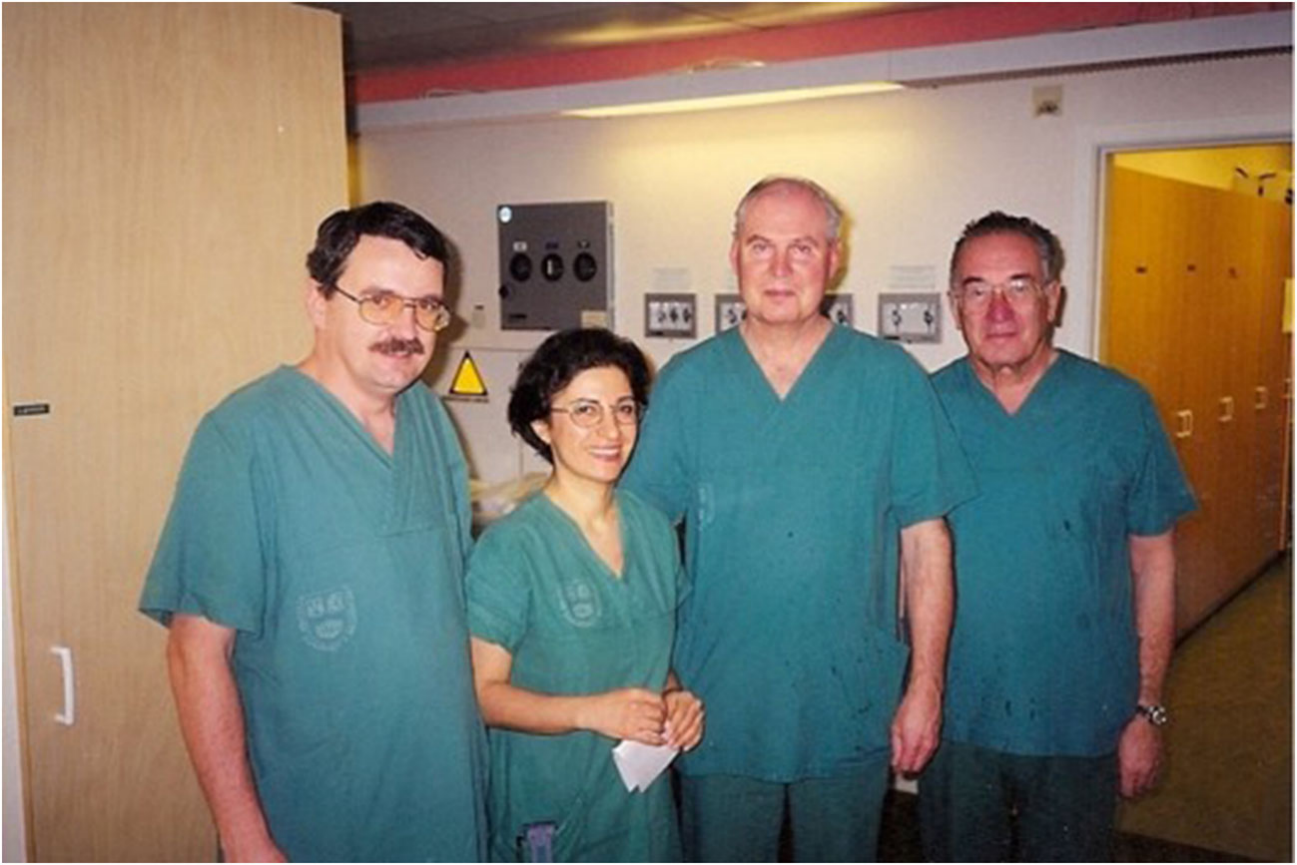
Uppsala 1997: Adolf Lukanović, Masoumeh Rezapour Isfahani, Ulf Ulmsten, Božo Kralj (from left to right)

During his career, Božo Kralj also served as the president of the Slovenian Association of Gynecologists and Obstetricians (1994–2005), president of the Italian Urogynecology Association (1989–1991), a member of the FIGO, a member of its scientific board (1997–2000) and its executive board (2003–2009), a member of the executive board of the EAGO, and an honorary member of the Gynecology Associations of Italy, Slovakia, and North Macedonia. Božo also broadened his knowledge as a Fulbright Scholar in the USA, an invited researcher in Long Beach and San Francisco, CA, as a British Council scholarship recipient in London, and at numerous symposia, conferences, and teaching workshops at home and abroad. He became a member of the European Academy of Sciences and Arts in 1992, and to the very end he remained an active member on many editorial boards of high-profile foreign and domestic scientific and professional journals. After retiring from the Gynecology and Obstetrics Division, he served as the dean of the University of Ljubljana Faculty of Health Sciences from 1998 to 2006, and thereafter as dean of the University of Novo Mesto Faculty of Health Sciences up until 2012 (Fig. 3).

Božo had a gift and decades of teaching experience, which he unselfishly shared with those of us he taught, advised, and trained. We are eternally grateful to him. Hidden behind his resolute charisma was his exceptional human warmth, which we gladly accepted with wonder and respect. The last of his significant contributions, which summarizes his many years of experience, was published in 2010 as a special editorial in IJGO entitled “Social and Therapeutic Challenges of Pelvic Floor Dysfunction.” It offers the still-relevant conclusion that the “rise in publications in journals on the topic of pelvic floor dysfunction suggests that the condition is increasingly recognized as an important area of women’s health.” Working together with him, we showed that “advances in understanding of the epidemiology and pathophysiology, as well as the development of subspecialists in the field, will lead to advances in prevention, management, and therapy of these important conditions.”

**Fig. 4:**
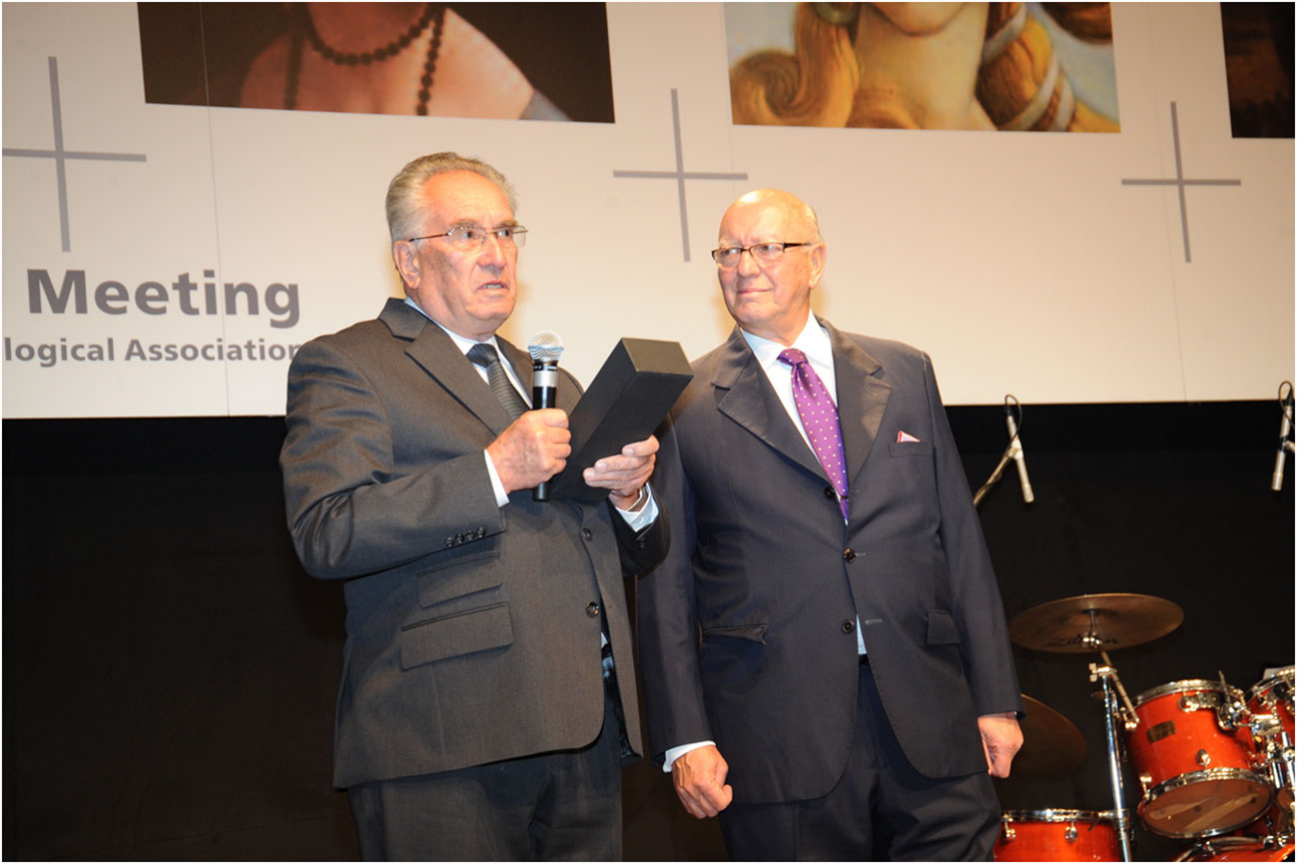
Božo Kralj receiving the International Urogynecological Association Lifetime Achievement Award in 2009, pictured here with Oscar Contreras Ortiz

**Fig. 5:**
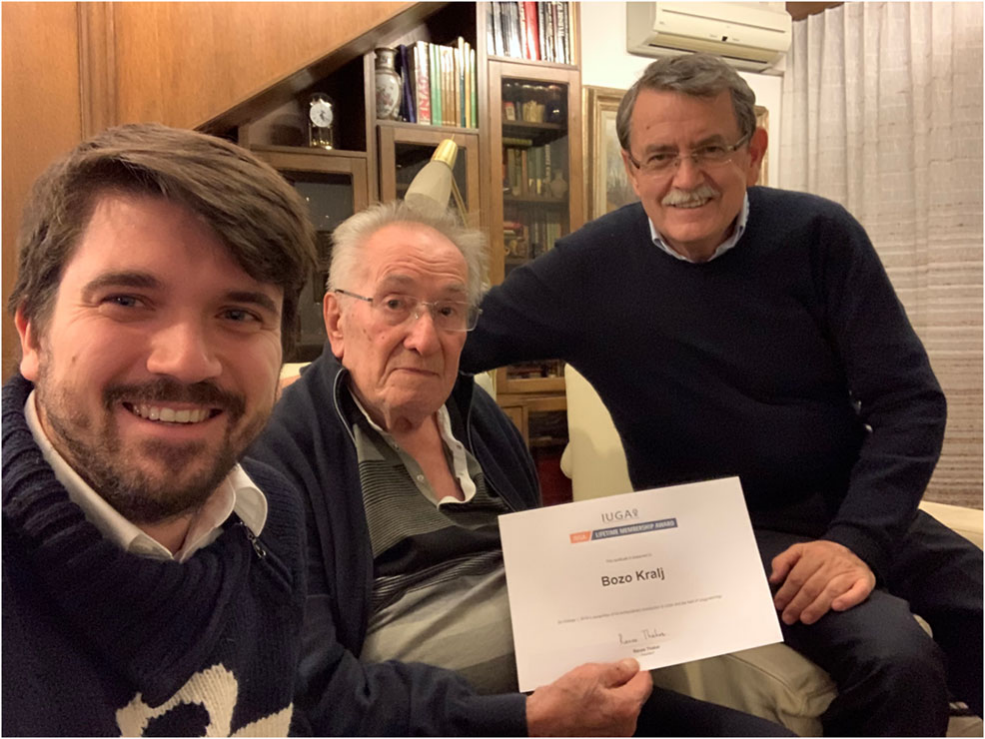
Božo Kralj receiving the International Urogynecological Association (IUGA) Lifetime Membership Award. Adolf and David Lukanović were authorized by the IUGA to present the award to him at his home in December 2019

For his life’s work and contribution to the international development of urogynecology he received the IUGA Lifetime Achievement Award, and for his contributions to developing international gynecology and obstetrics he was awarded the *Medaglia d’oro* (Gold Medal) of the Italian Association of Gynecologists and Obstetricians, both in 2009. In addition to numerous other awards, he was awarded the Yugoslav Order of Merit with Silver Rays in 1974, the City of Ljubljana Plaque in 1998, and the University of Ljubljana Gold Plaque in 2002 (Figs. 4, 5).

We will also remember Božo Kralj as a great man, an expert with wisdom and sound advice, a benefactor, and a fellow Rotarian. He was one of the first members of the Ljubljana Rotary Club, which was the first Rotary Club to be reestablished in southeastern Europe in 1990. He was endowed with an abundance of sparkling wit, with ever-surprising clarity and insight leavened with the humor that we now sorely miss. Božo created an openness and a positive attitude that left the freedom for professional growth and creativity to all who had the desire and interest to pursue it. He left us a rich legacy of knowledge as a man, a doctor, a colleague, and a friend. He managed to create a charismatic personality that will never fade.

Every loss of a beloved person is—for all of us, and especially for relatives of the deceased—one more proof that life is fleeting. We must rationally accept this supremacy of nature at the cost of the pain that saddens us and depletes our spirits, but at the same time ennobles us through recognition of the greatness of the immortality of nature. We accept this truth, dear Dr. Kralj!

*Ego plus quam feci, facere non possum,* said Cicero: “I can do no more than I have done.” You accomplished a great deal and we are thankful for all of your work.

